# Antibiotic-resistant pathogens in different patient settings and identification of surveillance gaps in Switzerland – a systematic review

**DOI:** 10.1017/S0950268819001523

**Published:** 2019-08-30

**Authors:** R. Fulchini, W. C. Albrich, A. Kronenberg, A. Egli, C. R. Kahlert, M. Schlegel, P. Kohler

**Affiliations:** 1 Division of Infectious Diseases and Hospital Epidemiology, Kantonsspital St. Gallen, St. Gallen, Switzerland; 2 Institute for Infectious Diseases, University Bern, Bern, Switzerland; 3 Clinical Microbiology Division, University Hospital Basel, Basel, Switzerland; 4 Applied Microbiology Research, Department of Biomedicine, University of Basel, Basel, Switzerland; 5 Division of Infectious Diseases and Hospital Epidemiology, Children's Hospital of Eastern Switzerland, St. Gallen, Switzerland

**Keywords:** Antibiotic resistance, Surveillance, Public health, Methicillin - S. aureus resistant to (MRSA), Gram-negative bacteria

## Abstract

The prevalence of antimicrobial resistance (AMR) varies significantly among different patient populations. We aimed to summarise AMR prevalence data from screening studies in different patient settings in Switzerland and to identify surveillance gaps. We performed a systematic review, searching Pubmed, MEDLINE, Embase (01/2000–05/2017) and conference proceedings for Swiss studies reporting on carbapenemase-producing *Enterobacteriaceae* (CPE), extended-spectrum beta-lactamases (ESBL), mobilised colistin-resistance, methicillin-resistant *Staphylococcus aureus* (MRSA) and vancomycin-resistant *Enterococci* (VRE) within different patient settings. We identified 2345 references and included 46 studies. For acute care patients, most screening data come from admission screenings, whereas AMR prevalence among hospitalised patients is largely unknown. Universal admission screenings showed ESBL-prevalences of 5–8% and MRSA-prevalences of 2–5%. For targeted screening, ESBL-prevalence ranged from 14–21%; MRSA-prevalence from 1–4%. For refugees, high ESBL (9–24%) and MRSA (16–24%) carriage rates were reported; returning travellers were frequently (68–80%) colonised with ESBL. Screening data for other pathogens, long-term care facility (LTCF) residents and pediatric populations were scarce. This review confirms high ESBL- and MRSA-carriage rates for risk populations in Switzerland. Emerging pathogens (CPE and VRE) and certain populations (inpatients, LTCF residents and children) are understudied. We encourage epidemiologists and public health authorities to consider these findings in the planning of future surveillance studies.

## Introduction

According to the World Health Organisation antimicrobial resistance (AMR) is one of the most concerning threats to modern medicine [1]. Surveillance has been formulated by the Centers for Disease Control and Prevention (CDC) as one of the four core actions among public health strategies against AMR [2]. Both national and international AMR surveillance programs are mainly based on phenotypic resistance data of bacterial isolates from clinical routine, without detailed information on patient characteristics or patient setting [3, 4]. However, AMR prevalence varies considerably between different patient populations, a fact which is not adequately represented in current surveillance systems. Examples for high-risk populations include patients transferred from abroad or residents of long-term care facilities (LTCF) [5, 6]. These high-risk groups could potentially act as reservoir for the distribution and spread of AMR within healthcare networks [7].

Knowing the prevalence of AMR among these populations might not only aid clinicians in their choice of empirical antibiotic treatment, but also support infection control specialists in prioritising prevention measures or guide public health authorities in planning future screening studies. In this systematic review, we aimed to summarise patient screening data for the most important antibiotic resistant pathogens within different patient settings in Switzerland, to identify screening gaps and to interpret these findings in the context of resistance data from neighbouring countries.

## Methods

### Aims

The primary study aims were (i) to summarise prevalence data on antibiotic resistance from studies performed in different patient settings in Switzerland between the year 2000 and 2017 and (ii) to identify gaps in surveillance for important patient populations. We focussed on studies reporting on carbapenemase-producing *Enterobacteriaceae* (CPE), extended-spectrum β-lactamase (ESBL)-producing or extended-spectrum cephalosporin resistant (ESC-R) *Enterobacteriaceae*, pathogens harbouring the mobilised colistin resistance (MCR)-gene, methicillin-resistant *Staphylococcus aureus* (MRSA) and vancomycin-resistant *Enterococci* (VRE). These five resistant pathogen groups (subsequently simply called pathogens) – accounting for almost two thirds of deaths caused by AMR in the US – were chosen based on their presumed importance for Switzerland [2].

The following settings are defined: acute/intensive care (i.e. universal and targeted admission screenings, other screenings), outpatients, LTCF and specific risk groups (i.e. refugees, travellers, people in contact with livestock, intravenous drug users [IVDU] and others). Studies in the paediatric population were analysed separately. This study is being reported according to the PRISMA guidelines [8].

### Design and study criteria

We performed a systematic literature search for studies meeting all of the following inclusion criteria: (i) studies (i.e. point-prevalence, cross-sectional and interventional studies, admission screenings, cohort and case-control studies) reporting prevalence data among specific patient populations; (ii) studies performed in Switzerland and (iii) majority of data collected after 1999. Studies on patients of all ages colonised with any of the five pathogen groups of interest at any body site (i.e. any screening method) were included.

The following exclusion criteria were applied: studies not including prevalence data (e.g. comments, reviews or editorials, case reports or outbreak reports without additional screenings); studies reporting AMR prevalence among clinical isolates but not among patients; studies focussing on animal or environmental samples; studies reporting only data on molecular epidemiology and studies including data from other countries, where the Swiss data were not presented separately.

The following microbiologic definitions had to be met:

- CPE: any *Enterobacteriaceae* with detection of a carbapenemase gene
- ESBL/ESC-R: any *Enterobacteriaceae* with non-susceptibility to a 3^rd^/4^th^-generation cephalosporine OR detection of an ESBL gene by polymerase chain reaction
- MCR: any pathogen with detection of a *mcr-*gene
- MRSA: *S. aureus* with non-susceptibility to oxacillin OR detection of *mecA* gene
- VRE: any *Enterococcus faecalis/faecium* with non-susceptibility to vancomycin OR documentation of *vanA*, *vanB* or *vanC* positivity

#### Search methods

A professional librarian performed a literature search in the Pubmed, MEDLINE and Embase databases (from 1 January 2000 to 5 May 2017). We used both medical subject headings and text word terms for AMR or each of the five key pathogens AND Switzerland (Supplements Table S1). Furthermore, conference proceedings of the European Congress of Clinical Microbiology and Infectious Diseases (ECCMID), 2015 to 2017, and the Joint Annual Meeting of the Swiss Society for Infectious Diseases (SSI), 2013–2017, were screened. Bibliographies of included records were screened for relevant articles. No language restrictions were applied. Studies which met inclusion criteria underwent full-text review. Abstract screening and full-text review were performed by two independent reviewers. Reviewers were not blinded for author names, institution names or the journal name.

#### Data extraction and management

The following data are abstracted from the selected studies: study design, study year(s), geographic location within Switzerland (French *vs.* German *vs.* Italian speaking regions), patient setting (see above), additional particular patient characteristics (if any), type of antibiotic resistance including resistance mechanism (if available) and screening sites. Every study was assigned a time period (i.e. 2000–2005; 2006–2011; 2012–2016), depending on when the majority of the study data were generated. All data were double entered by two independent reviewers and any discrepancies were resolved by consensus. Because of presumed heterogeneity between studies, no pooled prevalence estimate was calculated. Prevalence data were plotted by type of pathogen and study setting (this analysis was only performed for CPE, ESBL and MRSA because of the small number of studies for MCR-producers and VRE).

## Results

**Fig. 1.**
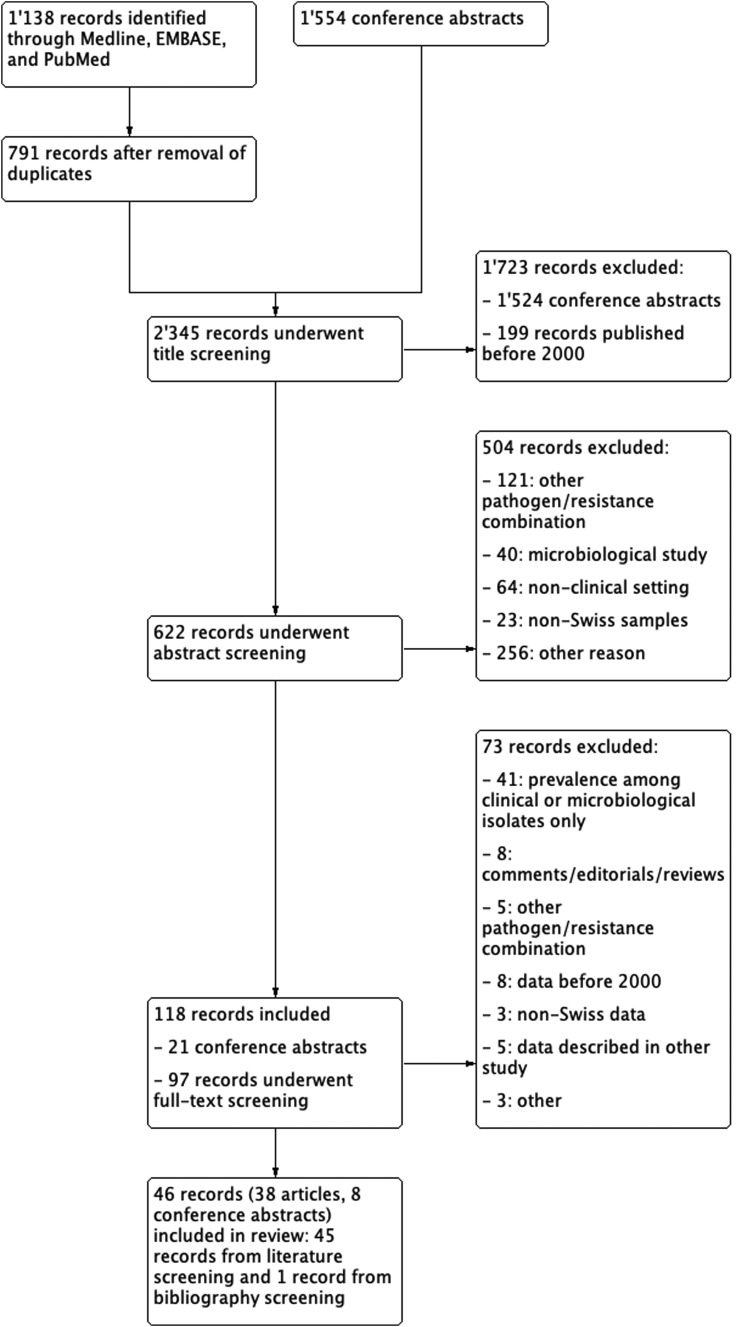
Results of literature search and flow-diagram.

**Fig. 2.**
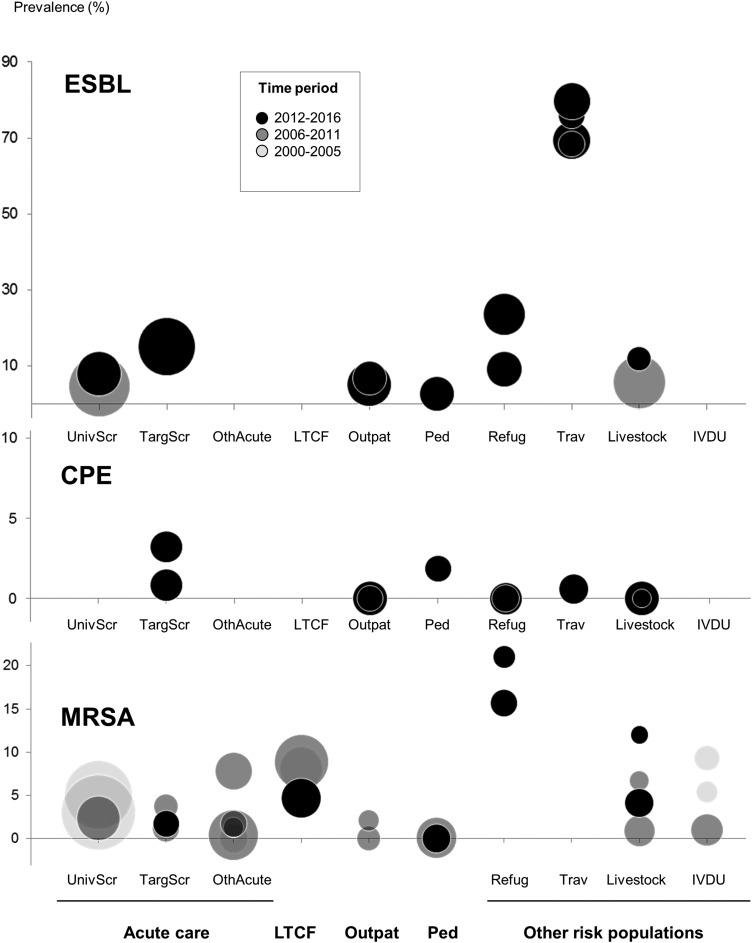
Prevalence data per patient setting and time period in Switzerland for ESBL, CPE and MRSA. Every circle represents a single study; circle diameter correlates with the study sample size. Please note the different scaling of the *y*-axis for the CPE, ESBL and MRSA. Acute/intensive care (UnivScr, Universal admission screening; TargScr, Targeted admission screening; OthAcute, Other inpatients); LTCF, Long-term care facility; Outpat, Outpatients; Ped, Paediatric patients; other risk populations (Refug, Refugees; Trav, Returning travellers; Livestock, people working in livestock industry; IVDU, Intravenous Drug Users).

We identified a total of 791 unique references and 1554 conference abstracts. One-hundred and eighteen records underwent full-text review and 46 records, thereof eight conference abstracts, were included in our review (Fig. 1). A descriptive summary of the available literature by patient setting is given below. Details of included studies, separated by Gram-negative and Gram-positive pathogens, are presented in Tables 1 and 2. For identification of surveillance gaps, studies are plotted in Figure 2, stratified by pathogen, patient setting and time period.

**Table 1.** Prevalence of antibiotic resistant Gram-negative pathogens among different patient populations in Switzerland

Author	Years	Setting	Comment	Study type	Region	Screening	Pathogens/mechanism	Prev	Number
CPE									
Kaspar [14]	2012/13	Acute care	Targeted: Hospitalisation abroad	Admission scr	East/Cent	N, I, R/S, Oth		0.9	2/235
Lemmenmeier [15]	2013–16	Acute care	Targeted: Hospitalisation abroad	Admission scr	East/Cent	R, Oth		3.2	7/217
Nüesch [39]	2012	Outpatients	GP pts	Cross sectional	Mixed/unk	S		0	0/291
Joao [29]	2015/16	Outpatients	HIV-infected pts	Cross sectional	Mixed/unk	S		0	0/101
Kuenzli [34]	2012/13	Other	Traveller to South Asia	Cohort	East/Cent	R	*E. coli* NDM-1	0.6	1/170
Zurfluh [38]	2014	Other	Meat processing company workers	Cross sectional	Mixed/unk	S	OXA-48	0.1	1/1086
Nüesch [39]	2012	Other	Meat processing company workers	Cross sectional	Mixed/unk	S		0	0/314
Erb [16]	2011–16	Other	Refugees	Admission scr	East/Cent	Unk		0	0/119
Piso [45]	2015	Other	Refugees	Cross sectional	Mixed/unk	R, U		0	0/241
Kraemer [41]	2015	Other	Pig farmers	Cross sectional	Mixed/unk	S		0	0/25
Moulin [51]	2016	Ped (in)	Neonatal ICU	Outbreak	West	S	*K. pneumoniae* OXA-48	1.9	2/108
ESBL or resistance to extended-spectrum cephalosporins									
Martinez [9]	2014–15	Acute care	Universal: medical/surgical ICU	Admission scr	East/Cent	R		7.9	24/302
Pasricha [10]	2010	Acute care	Universal	Admission scr	West	R	76% *E. coli*	4.8	51/1072
Nemeth [18]	2009–11	Acute care	Targeted: Hospitalisation abroad	Admission scr	East/Cent	N, P, I, Oth		13.9	36/259
Kaspar [14]	2012/13	Acute care	Targeted: Hospitalisation abroad	Admission scr	East/Cent	N, I, R/S, Oth		17.0	40/235
Lemmenmeier [15]	2013–16	Acute care	Targeted: Hospitalisation abroad	Admission scr	East/Cent	R, Oth		15.2	33/217
Fankhauser [17]	2006	Mixed	Targeted: Hospitalisation abroad	Admission scr	West	R	*Enterobacteriaceae*	18.0	22/124
Nüesch [28]	2012	Outpatients	GP pts	Cross sectional	East/Cent	S	Mostly *E. coli*, CTX-M	5.2	15/291
Joao [29]	2015/16	Outpatients	HIV-infected pts	Cross sectional	Mixed/unk	S	*E. coli*, mostly CTX-M-15	6.9	7/101
Vuichard [49]	2014–16	Other	Pregnant women	Cross sectional	East/Cent	R, V	*E. coli*	3.2	6/190
Geser [40]	2010	Other	Meat processing company workers	Cross sectional	Mixed/unk	S	*E. coli,* mostly CTX-M	5.8	34/586
Kraemer [41]	2015	Other	Pig farmers	Cross sectional	Mixed/unk	S	*E. coli*, CTX-M	12.0	3/25
Kuenzli [36]	2015	Other	Pre-Travel South-East Asia	Cohort	Mixed/unk	Unk		0	0/147
Bernasconi [35]	2015	Other	Pre-Travel India	Cross sectional	Mixed/unk	S	*E. coli*	7.9	3/38
Pires [37]	2014/15	Other	Pre-Travel India	Cohort	Mixed/unk	S		10.0	4/40
Uckay [50]	2009–11	Other	HCW on orthopedic wards	Cohort	West	R		12.2	6/41
Erb [16]	2011–16	Other	Refugees	Cross sectional	East/Cent	Unk		9.2	11/119
Piso [45]	2015	Other	Refugees	Cross sectional	Mixed/unk	R, U	*E. coli*	23.7	57/241
Kuenzli [34]	2012/13	Other	Post-Travel South-East Asia	Cohort	East/Cent	R	Mostly *E. coli*	69.4	118/170
Pires [37]	2014/15	Other	Post-Travel India	Cohort	Mixed/unk	S		76.0	31/40
Bernasconi [35]	2015	Other	Post-Travel India	Cross sectional	Mixed/unk	S	*E. coli*, CTX-M	68.4	26/38
Kuenzli [36]	2015	Other	Post-Travel South-East Asia	Cohort	Mixed/unk	Unk		79.6	117/147
Moulin [51]	2016	Ped (in)	Neonatal intensive care	Outbreak	West	S	*K. pneumoniae*	2.8	3/108^a^
MCR									
Joao [29]	2015/16	Outpatients	HIV-infected pts	Cross sectional	Mixed/unk	S	*E. coli* MCR-1	1.0	1/101
Zurfluh [30]	2016	Outpatients	GP pts	Cross sectional	East/Cent	S		0	0/53
Bernasconi [35]	2015	Other	Pre-travel India	Cross sectional	Mixed/unk	S		0	0/38
Bernasconi [35]	2015	Other	Post-travel India	Cross sectional	Mixed/unk	S	MCR-1	2.6	1/38
Zurfluh [30]	2016	Other	Meat processing company workers	Cross sectional	Mixed/unk	S		0	0/1091

Prev, prevalence (in %); Pt, patient; N, nasal; P, pharyngeal or throat; I, inguinal or groin; S, stool or faecal; R, rectal or perineal; U, urine; V, vaginal, Oth, others sites (depending on clinical picture); Unk, unknown; Ped (in), pediatric inpatients; Scr, screening; Cent, central; GP, general practitioner; HCW, healthcare worker.

a Result from contact screening, in addition three neonates were involved in the actual outbreak.

**Table 2.** Prevalence of antibiotic resistant Gram-positive pathogens among different patient populations in Switzerland

Author	Years	Setting	Comment	Study type	Region	Screening site(s)	Prev	Number
MRSA								
Harbarth [12]	2004–06	Acute care	Universal: 94% of all surgical pts	Admission scr	West	N, R, Oth	5.1	515/10 193
Pasricha [13]	2010	Acute care	Universal: 86% of internal medicine pts	Admission scr	West	N, I	2.4	41/1740
Witteck [19]	2008–09	Acute care	Targeted: High risk pts	Admission scr	East/Cent	N, P, A/I, Oth	3.7	6/161
Nemeth [18]	2009–11	Acute care	Targeted: Hospitalisation abroad	Admission scr	East/Cent	N, P, I, Oth	1.2	3/259
Kaspar [14]	2012/13	Acute care	Targeted: Hospitalisation abroad	Admission scr	East/Cent	N, I, R/S, Oth	1.7	4/235
Harbarth [11]	2003	Mixed	Universal: focus on CA-MRSA	Admission scr	West	N, I, Oth	3.0	428/14 253
Huttner [21]	2005–06	Acute care	Pts 30 days before colorectal resection	Cohort	West	N, R, Oth	9.3	18/194
Landelle [22]	2007	Acute care	All patients	Discharge scr	West	N, I	7.8	70/898
Buhlmann [20]	2005–06	Acute care	Patients at risk including admission scr	Other	East/Cent	N, I, Oth	1.7	4/232
Bächli [23]	2008–14	Acute care	Contacts of MRSA pts	Other	East/Cent	Unk	0.4	12/3013
Valsesia [24]	2009	Acute care	Contacts of MRSA pts	Other	East/Cent	N	1.3	1/80
Sax [25]	2001/03	LTCF	Universal scr	Admission scr	West	N, R, Oth	8.1	131/1621
Bellini [26]	2010/11	LTCF	Pts from 104 different nursing homes	RCT	West	N, I, Oth	8.9	366/4108
Héquet [27]	2015	LTCF	Pts from 33 different nursing homes	Cross sectional	West	N, P, I	4.7	54/1150
Marschall [31]	2004–05	Outpatients	Pregnant women from Ex-Yugoslavia	Cross sectional	East/Cent	N, V	0	0/152
Zimmerli [32]	2006	Outpatients	Dentist pts colonised with *S. aureus*	Cross sectional	East/Cent	N, P	1.0	2/210
Fleisch [46]	2002	Other	IVDU	Cross sectional	East/Cent	N	10.3	23/224
Fleisch [48]	2005	Other	IVDU	Cross sectional	East/Cent	N	5.4	6/111
Colombo [47]	2008–09	Other	IVDU	Cross sectional	East/Cent	N, P, Oth	1.0	5/497
Fleisch [48]	2002	Other	Healthcare workers in care of IVDU	Cross sectional	East/Cent	N	0	0/80
Valsesia [24]	2009	Other	Healthcare workers in care of MRSA pts	Other	East/Cent	N	0	0/202
Huber [43]	2009	Other	Slaughterhouse workers	Cross sectional	Mixed/unk	N	0	0/179
Huber [43]	2009	Other	Veterinarians	Cross sectional	Mixed/unk	N	3.0	4/133
Wettstein [44]	2012	Other	Veterinarians	Cross sectional	Mixed/unk	N	4.1	14/340
Piso [45]	2015	Other	Refugees	Mixed	Mixed/unk	N, P, I	15.7	41/261
Erb [16]	2011–16	Other	Refugees	Admission scr	East/Cent	Unk	21.0	25/119
Oppliger [42]	2008–09	Other	Pig farmers/veterinarians	Cross sectional	West	N	6.7	5/75
Huber [43]	2009	Other	Pig farmers	Cross sectional	Mixed/unk	N	0	0/148
Kraemer [41]	2015	Other	Pig farmers	Cross sectional	Mixed/unk	N	12.0	3/25
Heininger [52]	2006	Ped (in)	All patients	Admission scr	Mixed/unk	N	0.1	1/1337
Johnson [53]	2014/15	Ped (in)	Universal scr (64%) of all pediatric pts	Admission scr	East/Cent	N	0	0/340
Valsesia [24]	2009	Ped (in)	Contacts of MRSA pts	Other	East/Cent	N	0	0/10
VRE								
Voide [88]	2010–11	Outpatients	Pts hospitalised during VRE outbreak	Discharge scr	West	R	0	0/203

Prev, prevalence (in %); Pt, patient; N, nasal; P, pharyngeal or throat; A, axillary; I, inguinal or groin; S, stool or faecal; R, rectal or perineal; U, urine; V, vaginal, Oth, others sites (depending on clinical picture); Unk, unknown; Ped (in), pediatric inpatients; Scr, screening; Cent, central; LTCF, long-term care facility; CA, community-acquired; IVDU, intravenous drug users; RCT, randomised controlled trial.

### Acute care

#### Universal screenings

Data from universal hospital admission screenings (i.e. screening of all patients upon hospital admission) are available for ESBL and MRSA. For ESBL, prevalences of 5% (2010) and 8% (2014/2015) have been reported [9, 10]. For MRSA, several older studies from the Geneva University Hospital showed prevalences of 3% (2003, mixed population) [11], 5% (2004–2006, surgical patients) [12] and 2% (2010, internal medicine) [13]. In the study from 2003, 13 of 428 MRSA isolates were classified as community-acquired (CA)-MRSA, thereof five with production of Panton-Valentine leukocidin (PVL) [11]. No data are available for other pathogens.

#### Targeted admission screenings

Targeted screenings (i.e. screening of high-risk patients upon admission, mostly those transferred from abroad) have shown low CPE prevalences between 1% (2012/2013) and 3% (2013–2016) [14, 15]. For ESBL, these numbers have ranged between 14% and 21% [14–18]. For MRSA, targeted screenings performed in the German-speaking part revealed low prevalence rates between 1% and 3% [14, 18–20].

#### Other screening studies

One study from 2005/2006 screened patients before colorectal surgery, whereof 9% were MRSA positive [21]. Another study found a MRSA prevalence of 7% among all discharged patients from a tertiary care hospital [22]. Both studies were performed in the Western part of Switzerland. Two more recent studies from the German part, evaluating MRSA prevalence among hospitalised contacts of known MRSA carriers, showed prevalence rates of only 0.4% (2008–2014) and 1.3% (2009) [23, 24].

### Long-term care facilities/geriatric hospitals

For CPE or ESBL-producers, no screening data are available from LTCFs. For MRSA, prevalence in geriatric patients upon hospital admission was reported to be 8% in 2001/2003 [25]. Screening of nursing home residents in 2010/11 showed a similar prevalence of 9%, with a decreasing trend over time (5% in a follow-up study from 2015) [26, 27]. All these studies were performed in the French speaking part of Switzerland.

### Outpatients

No CPE carriers were detected in two cross-sectional studies among primary care (2012) and HIV-infected patients (2016) [28, 29]. The same studies also assessed ESBL carriage, showing a prevalence of 5% and 7% [28, 29]. Two cross-sectional studies looked at MCR prevalence among outpatients. Whereas the one study among HIV patients found 1% to be colonised with an MCR-1 producing *E. coli* [29], the other study did not identify any cases [30]. Other screening studies among outpatients have shown MRSA prevalence rates of 0% (pregnant women from former Yugoslavia), 1% (dental care patients) and 2% (patients with skin infections) [31–33].

### Other specific risk groups

#### Returning travellers

Screening for CPE identified only 1 carrier of NDM-1 *E. coli* (0.6%) among returning travellers from South Asia (2012/2013) [34]. ESBL-carriage was much more common and ranged between 68 and 80% (2012–2015) [34–37]. For MCR, 1 out of 38 (3%) travellers to India was found to have acquired an MCR-1 harbouring *E. coli* during travel [35].

#### People in contact with livestock

A dedicated group of Swiss investigators has studied the presence of resistant pathogens among workers of a meat processing factory. In one study, one out of more than 1000 (0.1%) workers was colonised with an OXA-48-producing *E. coli* (2014), whereas no CPE were detected in a similar study 2 years earlier [38, 39]. For ESBL, prevalence was 6% in the same population (2010) [40]; a study from 2016 regarding colonisation with MCR-producers revealed no positive sample [30]. Among pig farmers, no CPE were detected in 2015 whereas ESBL-prevalence was relatively high at 12% [41]. MRSA prevalence among pig farmers was 6.6% in 2008 [42], 0% in 2009 [43] and 12% in 2015 [41]. No MRSA was detected among slaughterhouse workers (2009), whereas 3% and 4% of screened veterinarians were MRSA-positive (2009 and 2012) [42–44].

#### Refugees

Two studies have assessed carriage of resistant pathogens among refugees. One study which screened asylum seekers presenting at a tertiary care hospital (2014/2015) showed an MRSA-prevalence of 21%, whereas ESBL- and CPE-prevalence was 9% and 0%, respectively [16]. Another cross-sectional study (2015) performed in four refugee centres found a MRSA-prevalence of 16%, ESBL-prevalence of 24% and again no CPE carriers [45].

#### Intravenous drug users

Several studies have looked at MRSA prevalence among IVDU. Whereas prevalence was as high as 10% around the year 2000 [46], the number dropped to 5.4% in 2005 and to 1% in 2008/2009 [47, 48].

#### Other adult populations

For ESBL, prevalence varies greatly by risk population: 3% (6/190) in pregnant women [49]; 0–10% in individuals before travel [35–37] and 12% among health-care workers [50]. The MRSA prevalence rate among healthcare workers in charge of MRSA patients was 0% (2002 and 2009) [24, 48].

### Pediatric population

During an outbreak of ESBL-producing *Klebsiella pneumoniae* in a neonatal intensive care unit 108 patients were screened, whereof six were positive. Unexpectedly, 2% were also positive for OXA-48 producing *K. pneumoniae* [51]. For MRSA, admission screening of high-risk patients found prevalence rates of 0.1% (2006) and 0% (2014/2015) [52, 53]. Another study from 2009 screening hospitalised contacts of pediatric MRSA patients confirmed this very low prevalence [24].

## Discussion

We comprehensively summarised the available literature on AMR screening data with regard to different patient settings in Switzerland. Data in this review confirm the high prevalence of ESBL among patients transferred from abroad and returning travellers; refugees are at high risk for both ESBL- and MRSA-carriage. In general, little information is available from LTCFs and the pediatric population; prevalence data on emerging pathogens such as CPE and VRE are scarce.

AMR is primarily a concern in acute and intensive care patients due to severe underlying illnesses, antibiotic pre-treatment and medical interventions predisposing for hospital-acquired infections [54–57]. According to a nation-wide survey in Swiss healthcare facilities, 83% of institutions are currently performing targeted admission screenings for multidrug-resistant pathogens. Considerable heterogeneity exists regarding target populations and screening methods [58]. Data from targeted admission screenings in Switzerland are comparable to numbers in Germany, where similar carriage rates among patients transferred from abroad for resistant Gram-negatives (13% *vs.* 14–17% in our review) and for MRSA (4% *vs*. 1–4% in our review) were found [6]. ESBL-prevalence from universal admission screening was 8% in a study included in our review, which is comparable to the 10% prevalence found in a German study [9, 59]. One of the most urgent antibiotic resistant threats, coming along with increased morbidity and mortality in infected patients, are CPE [60]. Looking at neighbouring countries, CPE prevalence in acute care settings varies considerably. Prevalence was reported to be as low as 0.1% upon hospital admission in Germany [61] and 0.4% among hospitalised acute care patients in southern France in 2012 [62]. In Italy, 8% of clinical *Enterobacteriaceae* isolates from inpatients were carbapenem-resistant in 2013 [63]. In Switzerland, according to national laboratory-based surveillance data, absolute numbers of CPE have been increasing in recent years, with OXA-48 being the most commonly reported carbapenemase followed by *K. pneumoniae* carbapenemase [64]. The fact that only 64% of Swiss healthcare institutions are actually performing targeted CPE admission screenings is worrisome [58]. Except from admission screenings, no prevalence data exist from Swiss acute care hospitals for these primarily nosocomial pathogens. However, modelling studies for the English hospital networks have shown that, because of the high number of patient transfers within the own healthcare network, the absolute risk of CPE introduction is higher for patients transferred within than those transferred from outside these networks, even if the local CPE prevalence is low [65]. In accordance with the conclusions drawn by the authors of this study, we think that further studies are needed to evaluate if CPE emergence in Switzerland is merely due to an increase of imported cases or if patients are increasingly acquiring CPE in Swiss hospitals. Regarding VRE, no screening studies have been published until date of this literature search. Of note, VRE have lately become a pressing issue in Switzerland, with multiple outbreaks having been reported within and between healthcare facilities [66, 67]. It remains to be seen if VRE will become endemic in this patient setting as it has for certain regions in Germany [68].

An important finding of our review is the scarcity of resistance data from Swiss LTCFs. This is concerning, because (i) LTCFs have been recognised as highly endemic settings for resistant pathogens already more than two decades ago [5, 69] and (ii) a recent laboratory-based study from Swiss LTCFs showed a clear increase in *E. coli* isolates resistant to extended-spectrum cephalosporins over the last decade [70]. Looking at neighbouring countries, data from LTCFs in Italy show that the prevalence of ESBL-producers and CPE is as high as 64% and 6%, respectively [71, 72]. A point prevalence study from 2015 performed in an Austrian LTCF reported a prevalence of 13% for ESBL, whereas no CPE were found [73]. In Germany, data from 2012 among nursing home residents showed a prevalence of 27% for ESBL, 9% for MRSA and 3% for VRE [74]. Only a minority of hospitals in Switzerland are routinely screening patients residing in LTCFs upon hospital admission [58]. Whether this strategy misses a relevant number of patients carrying resistant pathogens is currently unknown.

Similar to our data, a recently published review on AMR among European migrants found a high prevalence of MRSA (8%) and resistant Gram-negative bacteria (27%), concluding that living conditions, access to healthcare and AMR detection should be improved in this high-risk population [75]. In line with our findings, data from Germany suggest that ESBL-colonisation seems to be common among refugees, whereas CPE are rarely detected [76]. Data from other countries as well as a recent review confirm the high prevalence of particularly resistant Gram-negative pathogens among returning travellers [77–80]. None of the studies in our review performed MRSA screening among returning travellers. However, a multicentre study from different European cities – including a Swiss study centre – reported an MRSA prevalence of 14% (2019) among returning travellers with skin and soft tissue infections, with Latin America as travel destination being a risk factor for MRSA carriage. A large proportion of these isolates were PVL-producing CA-MRSA [81]. Interestingly, although MRSA rates are generally decreasing in Switzerland [82], the prevalence among pig farmers – who are likely to be colonised with livestock-associated MRSA – has increased between 2009 and 2015. This finding is in line with reports from Germany, showing increasing trends for LA-MRSA while overall MRSA rates are declining [83, 84]. Until now, CPE and MCR are not commonly detected in these settings in Switzerland. Again, no data exist for VRE.

Studies on AMR screening from the pediatric population are rare in Switzerland. National laboratory-based surveillance data suggest that the prevalence of ESBL and MRSA among clinical isolates is similar to that of the adult population [82, 85, 86]. Of note, the unexpected finding of two neonates carrying OXA-48-producing *K. pneumoniae* in Switzerland is worrying and warrants further study [51]. Similar to adult refugees, pediatric refugees are at particular risk of carrying resistant pathogens, as shown recently in two German studies [76, 87].

Our study has some limitations. First, the heterogeneity among included studies (e.g. definitions of risk for targeted admission screenings, different screening sites) precluded us from calculating a pooled prevalence or from statistically assessing trends over time. In this context it is particularly important to note that clinical microbiology breakpoints have changed during the observed time period. Second, although we performed a thorough literature search we might have missed studies, especially those only presented as abstracts in conferences which were not on our screening list. Third, some of the most recent publications were only available in an abstract form. Nonetheless, we included these studies as we would have otherwise missed the most recent data in this rapidly changing field.

In conclusion, this review confirms data from other countries showing high prevalence of ESBL-carriage among patients transferred from abroad and returning travellers; refugees are at risk for both ESBL and MRSA. There is a scarcity of AMR prevalence data from Swiss LTCFs and the pediatric population. Also, screening data are generally scarce for emerging pathogens such as CPE or VRE, which often spread within and between acute care facilities. We encourage epidemiologists and public health authorities to consider these gaps in the planning of future surveillance studies.

## Supplementary material

For supplementary material accompanying this paper visit https://doi.org/10.1017/S0950268819001523. click here to view supplementary material
