# Supplementary material for: Antibiotic-resistant pathogens in different patient settings and identification of surveillance gaps in Switzerland – a systematic review
Source: Epidemiol Infect. 2019 Aug 30;147:e259. doi: 10.1017/S0950268819001523 (PMC6805757; doi:10.1017/S0950268819001523)
Supplement: Supplementary file 1 [file S0950268819001523sup001.docx]

**SUPPLEMENTS**

Table S1. Search strategies employed (for Embase Search, 5 May 2017).

| **No.** | **Query** | **Results** |
| --- | --- | --- |
| #4 | #1 AND #2 NOT ([animals]/lim NOT [humans]/lim) NOT [conference abstract]/lim | 707 |
| #3 | #1 AND #2 | 1008 |
| #2 | 'switzerland'/exp OR switzerland:ti,ab OR swiss:ti,ab | 83936 |
| #1 | 'antibiotic resistance'/exp OR 'methicillin resistant staphylococcus aureus'/exp OR 'vancomycin resistant  staphylococcus aureus'/exp OR 'linezolid resistant staphylococcus aureus'/exp OR 'methicillin resistant  staphylococcus aureus infection'/exp OR 'drug resistant tuberculosis'/exp OR 'vancomycin resistant  enterococcus'/exp OR 'carbapenem resistant enterobacteriaceae'/exp OR 'extended spectrum beta  lactamase'/exp OR ((antibiotic OR antibacterial OR antimicrobial OR 'bacterial drug' OR bacterial OR bacterium  OR 'beta lactam' OR cephalosporin OR ceftriaxone OR ceftazidime OR vanomycin OR carbapenem OR  doripenem OR imipenem OR meropenem OR ertapenem OR oxicillin OR methicillin OR meticillin) NEAR/3  (resistan* OR 'non susceptib*' OR unsusceptib* OR insensitiv* OR nonsensitiv* OR 'non sensitiv*')):ti,ab OR  ((bacterial OR microbial) NEAR/3 'drug resistan*'):ti,ab OR ((esbl* OR 'beta lacatmase') NEAR/3  enterobacteria*):ti,ab OR esbl:ti OR mrsa:ti OR ('antibiotic agent'/exp AND (resistan*:ti OR 'non susceptib*':ti  OR unsusceptib*:ti OR insensitiv*:ti OR nonsensitiv*:ti OR 'non sensitiv*':ti)) OR ('antibiotic agent'/exp/mj AND  (resistan*:ti,ab OR 'non susceptib*':ti,ab OR unsusceptib*:ti,ab OR insensitiv*:ti,ab OR nonsensitiv*:ti,ab OR  'non sensitiv*':ti,ab)) | 250540 |
